# Red blood cell distribution width and mortality risk in critically ill cardiovascular patients

**DOI:** 10.1016/j.heliyon.2023.e22225

**Published:** 2023-11-11

**Authors:** Shan Li, Wei Zhang, Xiao Liang

**Affiliations:** Department of Cardiology, Second Medical Center & National Clinical Research Center for Geriatric Diseases, Chinese PLA General Hospital, Beijing, China

**Keywords:** Red blood cell distribution width, All-cause mortality, Cause-specific mortality, Critical illness, Cardiovascular disease

## Abstract

**Background:**

The association between red blood cell distribution width (RDW) and mortality risk in critically ill cardiovascular patients has not been well studied.

**Objective:**

To examine the association between RDW and 30-day all-cause and cause-specific mortality in critically ill cardiovascular patients.

**Methods:**

This cohort study included 47,266 patients from the eICU database. RDW was categorized as <13.0 %, 13.0–13.4 %, 13.5–13.9 %, 14.0–14.4 %, 14.5–14.9 %, ≥15.0 %. Logistic regression model was used to estimate adjusted odds ratios (ORs), and log-linear regression model was used to examine absolute rate differences (RDs) in mortality risk. Cubic spline curve was used to explore the nonlinear association between changes in RDW and mortality.

**Results:**

A graded association between higher RDW and incremental risk of death was observed. Compared with RDW of <13.0 %, the adjusted odds ratios for all-cause mortality were 1.29 (95 % CI, 1.10 to 1.53) for RDW of 13.5–13.9 %, 1.57 (95 % CI, 1.33 to 1.85) for RDW of 14.0–14.4 %, 1.94 (95 % CI, 1.64 to 2.29) for RDW of 14.5–15.0 %, and 3.15 (95 % CI, 2.74 to 3.63) for RDW of ≥15.0 %. The absolute rate differences for RDW of 13.5–13.9 %, 14.0–14.4 %, 14.5–14.9 %, and ≥15.0 % indicated an additional 6, 13, 14, and 40 deaths per 1000 patients, respectively. RDW was associated with most, but not all, cause-specific deaths.

**Conclusion:**

RDW was strongly associated with all-cause mortality and most cause-specific mortality in critically ill cardiovascular patients. These findings underscore the importance of this readily available hematologic indicator in mortality risk stratification.

## Highlights

• Elevated RDW was associated with increased all-cause mortality in critically ill cardiovascular patients.
• RDW was associated with most, but not all, cause-specific deaths in critically ill cardiovascular patients.
• There was a nonlinear relationship between dynamic changes in RDW and all-cause mortality.
• These findings underscore the importance of this readily available indicator in mortality risk stratification.

## 1 Introduction

As the leading cause of global mortality and a major contributor to disability, cardiovascular disease (CVD) remains a major public health concern [1,2]. Over the past three decades, CVD mortality has continued to rise, from 12.1 million in 1990 to 18.6 million in 2019 [3]. The management of cardiovascular patients in the intensive care unit (ICU) is challenged by the heightened risk of mortality and morbidity. Valuable biomarkers are urgently needed for risk stratification to ensure that high-risk patients receive close monitoring and additional care, thereby reducing overall mortality and optimizing health economics.

Red cell distribution width (RDW) is a ubiquitous indicator included in a complete blood count that represents the heterogeneity of red cell volume [4]. A growing body of evidence suggests that elevated RDW is associated with an increased risk of adverse outcomes in a variety of physiological disorders, including heart disease [5,6], sepsis [7], metabolic disorders [8], cancer [9,10] and the global pandemic COVID-19 [11,12]. A recent proteomic analysis has further identified promising candidate biomarkers linking elevated RDW to excess mortality [13]. Previous studies have also shown that RDW is a robust prognosticator of all-cause mortality in the general critically ill population [14,15]. However, the predictive potential of RDW for mortality in critically ill cardiovascular patients undergoing contemporary therapy remains understudied. Moreover, it remains uncertain whether the mortality predictors found in the general cardiovascular population are equally applicable to critically ill cardiovascular patients. This is because their underlying risk factors for death, such as disease severity, cardiac comorbidities, prevalence of anemia, and nutritional status, are significantly different.

We investigated the association between RDW and 30-day mortality in critically ill cardiovascular patients using the large multicenter eICU database. The objectives of this study were threefold: first, to determine the association between RDW and all-cause mortality; second, to evaluate the impact of RDW change on all-cause mortality; and third, to examine the association between RDW and cause-specific mortality.

## 2 Methods

### 2.1 Data source

The eICU database is a telehealth program developed by the Massachusetts Institute of Technology in collaboration with Philips Healthcare [16]. The database contains electronic medical information from more than 300 ICUs in the U.S. between 2014 and 2015 and is used for extensive clinical research and teaching. This study was conducted in accordance with the Declaration of Helsinki. The study is exempt from institutional review board approval due to its retrospective design and lack of direct patient intervention. The Massachusetts Institute of Technology approved the use of the database and waived patient informed consent. We extracted data for individuals admitted to the ICU for cardiovascular reasons (n = 61,669). Exclusion criteria were (1) no RDW data available (n = 14,293), (2) age <18 years (n = 11), and (3) RDW <10 % or >30 % (n = 99). Finally, 47,266 individuals were included in the analysis.

### 2.2 Exposure

The primary exposure was RDW, which was categorized as <13.0 %, 13.0–13.4 %, 13.5–13.9 %, 14.0–14.4 %, 14.5–14.9 %, ≥15.0 %. RDW <13.0 %, the lowest risk category, was used as the reference category. To reduce the influence of RDW variability on the results, we analyzed baseline, mean, peak, and nadir RDW separately. 47,266 patients had a baseline measurement (93.2 % within 24 h of admission and 99.5 % within 3 days of admission), and 36,723 patients had at least 2 different RDW measurements. When only one measurement was available, baseline RDW was defined as mean, peak, or nadir RDW. If more than one measurement was available, the actual mean, peak, and nadir values were used.

### 2.3 Outcomes

The primary outcome was all-cause mortality and the secondary outcome was cause-specific mortality. Disease diagnoses were determined by International Classification of Diseases, 9th Revision codes. Cardiovascular disease was defined by ICD-9 codes 390 to 459 and 785. Specific diseases were defined as follows: acute myocardial infarction, ICD-9 code 410; congestive heart failure, ICD-9 code 428; cardiac arrest and arrhythmia, ICD-9 code 427; ischemic stroke, ICD-9 codes 433 to 434; intracerebral hemorrhage, ICD-9 code 431; subarachnoid hemorrhage, ICD-9 code 430; pulmonary embolism, ICD-9 code 415; deep vein thrombosis, ICD-9 code 453.4; aortic aneurysm and dissection, ICD-9 code 441; cardiogenic shock, ICD-9 code 785.5.

### 2.4 Covariates

The following potentially relevant factors were included in the models: demographics and baseline conditions, including age, sex, ethnicity, body mass index (BMI), mean blood pressure, heart rate, Glasgow Coma Scale (GCS), and Acute Physiology, Age and Chronic Health Evaluation (APACHE) score; laboratory measurements, including white blood cell count, hemoglobin, hematocrit, mean corpuscular volume (MCV), albumin, and creatinine; primary admission diagnosis, including acute myocardial infarction, congestive heart failure, cardiac arrest or ventricular arrhythmia, atrial or other arrhythmia, ischemic stroke, intracerebral hemorrhage, subarachnoid hemorrhage, pulmonary embolism or deep vein thrombosis, aortic aneurysm or dissection, cardiogenic shock, hypertension, and cardiomyopathy; previous comorbidities, including myocardial infarction, percutaneous coronary intervention, coronary artery bypass graft, chronic heart failure, atrial fibrillation, diabetes mellitus, hypertension, stroke or transient ischemic attack, chronic obstructive pulmonary disease, renal insufficiency, dementia, and cancer; major treatments, including mechanical ventilation, dialysis, red blood cell transfusion, anticoagulants, antiplatelet agents, and lipid-lowering agents.

### 2.5 Statistical analysis

Continuous variables are presented as mean (standard deviation) or median (interquartile range), and categorical variables are presented as numbers (proportions). Differences in baseline characteristics were compared using ANOVA, Kruskal-Wallis test, or chi-squared test. Missing data were imputed using the R package multiple imputation method of chained equations with 5 imputations (21.1 % for albumin, 15.6 % for mean corpuscular volume, 9.9 % for APACHE score, 4.9 % for BMI, 1.7 % for GCS, 0.2 % for white blood cell count). Statistical analyses were performed using R software (version 4.2.0, http://www.r-project.org) and EmpowerStats (http://www.empowerstats.com, X&Y Solutions, Inc., Boston, MA). Two-sided P values of <0.05 indicated statistical significance.

The association of RDW with all-cause mortality was examined using a logistic regression model. Risk estimates for patients with and without anemia were presented separately to account for the effect modification. Anemia was defined as hemoglobin <13 g/dL for men and Hb < 12 g/dL for women [17]. Linear trend was tested by entering the median value of each category of RDW as a continuous variable. A cubic spline curve based on the generalized additive model was used to intuitively visualize the association shape. The magnitude of absolute effects was quantified using a log-linear regression model with robust variance estimates, with results expressed as adjusted absolute risk differences with 95 % confidence intervals. The absolute risk difference was reported as the excess number of deaths per 1000 patients compared with the reference category. Dynamic changes in RDW were assessed by the difference between the last and first available RDW measurement in each patient who had at least two measurements. A cubic spline curve was used to explore the nonlinear relationship between changes in RDW and all-cause mortality, and 500 bootstraps were used to estimate the inflection point and 95 % CI. A time-varying model of the RDW was constructed and stratified by normal (11.5–14.5 %) and elevated (>14.5 %) RDW and survival status, with the cohort limited to the first 20 days of ICU admission (most measurements were completed during this period), covering 96.7 % of RDW measurements. Logistic regression model was also used to estimate the risk of 10 cause-specific deaths. The Bonferroni correction was applied to the 10 independent comparisons on the same data, with the level of statistical significance set at 0.005, and P values categorized as follows: <0.05 (suggestive of a trend), <0.005 (statistically significant), and <0.0001 (strong evidence).

Several sensitivity analyses were performed, including subgroup analyses to evaluate the interactions of RDW with age, sex, ethnicity, hematocrit, MCV, and mechanical ventilation on all-cause mortality; an analysis excluding deaths within the first 24 or 48 h of ICU admission to mitigate reverse causality; a complete case analysis to address the impact of missing covariate data; an analysis using the actual mean, peak, and nadir RDW in patients with more than one measurement; a cox proportional hazards model using ICU length of stay as time scale to account for potential modification of results by different statistical method; and finally, an analysis entering RDW and all of the covariates into a logistic regression model to determine independent predictors of all-cause mortality.

## 3 Results

### 3.1 Baseline characteristics

Of the 61,669 patients admitted for cardiovascular reasons, 47,266 had RDW available for analysis. Patients included in the analysis were older, more likely to be Caucasian, more likely to be admitted for cardiac surgery, and more likely to have a history of hypertension, prior myocardial infarction, or percutaneous coronary intervention than patients not included in the analysis (eTable 1 in the Supplement). The study cohort had a median age of 66.9 (SD 14.4) years with a slight male predominance (58.4 %) and was ethnically diverse with a majority of Caucasians (78.0 %). Individuals with a higher RDW were slightly older, had higher APACHE scores, a higher prevalence of congestive heart failure, cardiac arrest or ventricular arrhythmia, and cardiogenic shock, and a lower prevalence of acute myocardial infarction and stroke. There were 4070 all-cause deaths, representing 8.6 % of the cohort, with all-cause mortality increasing progressively with increasing RDW. The characteristics of patients in baseline RDW categories are shown in Table 1. The mean RDW values were 14.8 ± 2.1 %, 14.9 ± 2.1 %, 15.3 ± 2.4 %, and 14.6 ± 2.0 % for baseline, mean, peak, and nadir measurements, respectively (eFig. 1 in the Supplement).

**Table 1:** Baseline characteristics of individuals by RDW categories. Values are mean (standard deviation), median (inter-quartile range) or number (percentage). RDW, red blood cell distribution width. APACHE, acute physiology, age and chronic health evaluation.

RDW, %	<13.0	13.0–13.4	13.5–13.9	14.0–14.4	14.5–14.9	≥15	P value
N (%)	5684 (12.0)	6944 (14.7)	7130 (15.1)	6160 (13.0)	4922 (10.4)	16,426 (34.8)	
Age, years	61.4 ± 15.0	64.6 ± 14.1	66.8 ± 14.2	68.3 ± 14.0	69.0 ± 13.8	68.6 ± 14.3	<0.001
Male, n (%)	3751 (66.0)	4502 (64.8)	4417 (61.9)	3524 (57.2)	2765 (56.2)	8659 (52.7)	<0.001
Ethnicity							<0.001
Caucasian, n (%)	4632 (81.5)	5704 (82.1)	5727 (80.3)	4840 (78.6)	3786 (76.9)	12,157 (74.0)	
Other/unknown, n (%)	1052 (18.5)	1240 (17.9)	1403 (19.7)	1320 (21.4)	1136 (23.1)	4269 (26.0)	
Heart rate, bpm	96 ± 31	92 ± 31	91 ± 32	91 ± 32	91 ± 33	97 ± 32	<0.001
Mean blood pressure, mmHg	85 ± 42	86 ± 42	89 ± 43	92 ± 43	93 ± 43	94 ± 43	<0.001
Body mass index, kg/m^2^	28.5 ± 5.9	29.0 ± 6.2	29.3 ± 6.6	29.3 ± 6.9	29.5 ± 7.2	29.6 ± 8.0	<0.001
Severity score							
Glasgow coma scale	12 ± 4	13 ± 4	13 ± 4	13 ± 3	13 ± 3	13 ± 4	<0.001
APACHE score	51 (37–68)	52 (37–68)	52 (38–68)	53 (38–70)	54 (40–71)	57 (41–75)	<0.001
Laboratory measurement							
White blood cell, × 10^9^/L	10.3 ± 5.2	10.8 ± 5.3	10.8 ± 5.1	10.9 ± 5.1	11.1 ± 5.0	12.3 ± 5.6	<0.001
Hemoglobin, g/dL	10.6 ± 2.2	11.4 ± 2.2	11.7 ± 2.2	12.0 ± 2.1	12.0 ± 2.2	11.6 ± 2.2	<0.001
Hematocrit, %	31.8 ± 6.4	34.1 ± 6.3	35.2 ± 6.2	36.0 ± 6.1	36.1 ± 6.2	35.3 ± 6.2	<0.001
Mean corpuscular volume, fL	91.6 ± 6.3	91.2 ± 6.0	90.6 ± 5.9	90.6 ± 5.9	90.2 ± 5.9	89.1 ± 6.5	<0.001
Albumin, g/L	28.9 ± 6.6	30.7 ± 5.8	31.3 ± 5.6	31.3 ± 5.8	31.7 ± 5.8	30. ±6.3	<0.001
Creatinine, mg/dL	1.1 (0.4–9.7)	1.0 (0.5–10.0)	1.0 (0.4–9.3)	1.0 (0.4–10.0)	1.0 (0.4–9.0)	1.0 (0.5–10.1)	<0.001
Primary diagnosis							
Acute myocardial infarction, n (%)	929 (16.3)	1176 (16.9)	1087 (15.2)	820 (13.3)	481 (9.8)	1141 (6.9)	<0.001
Ischemic stroke, n (%)	788 (13.9)	835 (12.0)	832 (11.7)	626 (10.2)	435 (8.8)	1098 (6.7)	<0.001
Intracerebral hemorrhage or subarachnoid hemorrhage, n (%)	470 (8.3)	452 (6.5)	429 (6.0)	317 (5.1)	243 (4.9)	490 (3.0)	<0.001
Congestive heart failure, n (%)	132 (2.3)	223 (3.2)	356 (5.0)	455 (7.4)	536 (10.9)	3065 (18.7)	<0.001
Cardiac arrest or ventricular arrhythmia, n (%)	360 (6.3)	465 (6.7)	497 (7.0)	444 (7.2)	413 (8.4)	1886 (11.5)	<0.001
Atrial or other arrhythmia, n (%)	280 (4.9)	352 (5.1)	444 (6.2)	383 (6.2)	347 (7.0)	1437 (8.7)	<0.001
Hypertension, n (%)	132 (2.3)	196 (2.8)	198 (2.8)	201 (3.3)	132 (2.7)	497 (3.0)	0.037
Cardiac surgery, n (%)	1126 (19.8)	1398 (20.1)	1370 (19.2)	1220 (19.8)	967 (19.6)	2451 (14.9)	<0.001
Cardiogenic shock, n (%)	29 (0.5)	33 (0.5)	47 (0.7)	40 (0.6)	57 (1.2)	218 (1.3)	0.012
Cardiomyopathy, n (%)	24 (0.4)	29 (0.4)	37 (0.5)	47 (0.8)	43 (0.9)	241 (1.5)	<0.001
Pulmonary embolism or deep venous thrombosis, n (%)	195 (3.4)	243 (3.5)	227 (3.2)	211 (3.4)	182 (3.7)	637 (3.9)	0.127
Aortic aneurysm or dissection, n (%)	110 (1.9)	156 (2.2)	175 (2.5)	187 (3.0)	138 (2.8)	341 (2.1)	<0.001
Pre-admission comorbidities							
Hypertension, n (%)	3346 (58.9)	4179 (60.2)	4229 (59.3)	3638 (59.1)	2878 (58.5)	9445 (57.5)	0.004
Stroke or TIA, n (%)	594 (10.5)	800 (11.5)	851 (11.9)	737 (12.0)	601 (12.2)	2130 (13.0)	<0.001
Prior myocardial infarction, n (%)	823 (14.5)	1050 (15.1)	1046 (14.7)	845 (13.7)	668 (13.6)	2016 (12.3)	<0.001
Percutaneous coronary intervention, n (%)	601 (10.6)	802 (11.5)	783 (11.0)	681 (11.1)	482 (9.8)	1538 (9.4)	<0.001
Coronary artery bypass grafting, n (%)	515 (9.1)	651 (9.4)	645 (9.0)	478 (7.8)	377 (7.7)	1044 (6.4)	<0.001
Chronic heart failure, n (%)	1301 (22.9)	1538 (22.1)	1453 (20.4)	1164 (18.9)	910 (18.5)	2968 (18.1)	<0.001
Atrial fibrillation, n (%)	980 (17.2)	1127 (16.2)	1033 (14.5)	820 (13.3)	640 (13.0)	2046 (12.5)	<0.001
Diabetes mellitus, n (%)	723 (12.7)	836 (12.0)	935 (13.1)	753 (12.2)	625 (12.7)	2230 (13.6)	0.013
Chronic obstructive pulmonary disease, n (%)	688 (12.1)	831 (12.0)	887 (12.4)	787 (12.8)	604 (12.3)	2153 (13.1)	0.133
Renal insufficiency, n (%)	999 (17.6)	1111 (16.0)	1025 (14.4)	780 (12.7)	595 (12.1)	1948 (11.9)	<0.001
Dementia, n (%)	98 (1.7)	171 (2.5)	155 (2.2)	169 (2.7)	136 (2.8)	421 (2.6)	<0.001
Cancer, n (%)	927 (16.3)	855 (12.3)	760 (10.7)	637 (10.3)	500 (10.2)	1755 (10.7)	<0.001
Theraputics							
Mechanical ventilation, n (%)	2136 (37.6)	2017 (29.0)	1709 (24.0)	1355 (22.0)	963 (19.6)	3418 (20.8)	<0.001
Dialysis, n (%)	299 (5.3)	316 (4.6)	289 (4.1)	231 (3.8)	158 (3.2)	532 (3.2)	<0.001
Red blood cell transfusion, n (%)	262 (4.6)	210 (3.0)	145 (2.0)	101 (1.6)	71 (1.4)	272 (1.7)	<0.001
Anticoagulants, n (%)	1259 (22.1)	1620 (23.3)	1695 (23.8)	1376 (22.3)	1073 (21.8)	3509 (21.4)	<0.001
Antiplatelet agents, n (%)	1374 (24.2)	1965 (28.3)	2077 (29.1)	1773 (28.8)	1437 (29.2)	4376 (26.6)	<0.001
Lipid-lowering agents, n (%)	1674 (29.5)	2371 (34.1)	2555 (35.8)	2182 (35.4)	1737 (35.3)	5288 (32.2)	<0.001
Length of stay, days	3.9 (2.1–6.1)	4.0 (2.2–6.4)	4.2 (2.3–7.0)	4.6 (2.6–7.2)	4.9 (2.8–8.0)	5.4 (3.0–9.1)	<0.001
All-cause death, n (%)	249 (4.4)	354 (5.1)	414 (5.8)	432 (7.0)	422 (8.6)	2199 (13.4)	<0.001

### 3.2 Association between RDW and all-cause mortality

**Fig. 1:**
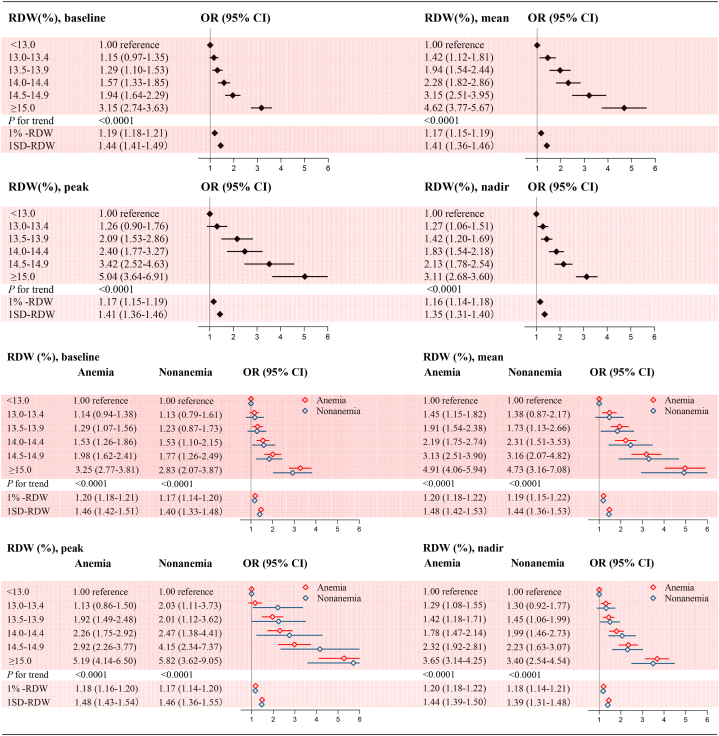
Adjusted odds ratios for all-cause mortality by categorical RDW Model was adjusted for all predefined covariates. RDW, red blood cell distribution width. SD, standard deviation. OR, odds ratio. CI, confidence interval.

**Fig. 2:**
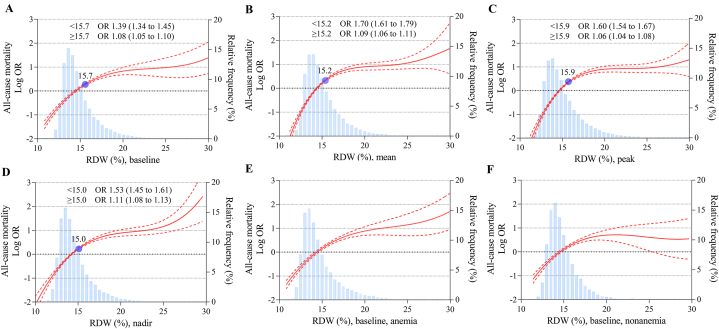
Adjusted odds ratios for all-cause mortality by continuous RDW Model was adjusted for all predefined covariates. (A) baseline RDW, (B) mean RDW, (C) peak RDW, (D) nadir RDW, (E) baseline RDW in patients with anemia, and (F) baseline RDW in patients without anemia. RDW, red blood cell distribution width. OR, odds ratio. CI, confidence interval.

**Fig. 3:**
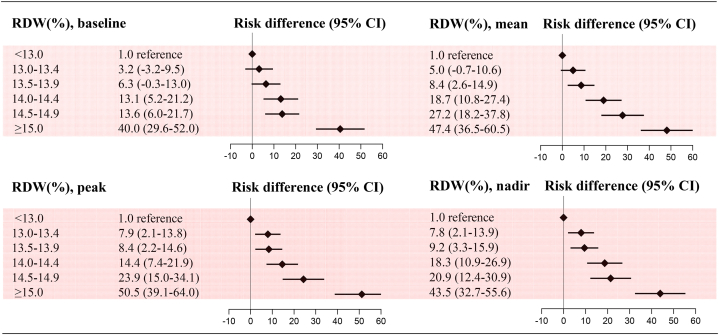
Adjusted risk differences (per 1000 patients) for all-cause mortality Model was adjusted for all predefined covariates. RDW, red blood cell distribution width. RD, risk difference. CI, confidence interval.

After adjustment for potential confounders, a stepwise and graded association between RDW and all-cause mortality was observed. Patients with a baseline RDW ≥15.0 % had a multifactorially adjusted odds ratio of 3.15 (95 % CI, 2.74–3.63) compared with those with RDW <13.0 %. Notably, patients with RDWs of 13.5–13.9 % and 14.0–14.4 % (generally considered the normal range) also had increased odds ratios of 1.29 (95 % CI, 1.10–1.53) and 1.57 (95 % CI, 1.33–1.85), respectively. When RDW was examined as a continuous variable, the risk of all-cause mortality increased by 19 % (OR 1.19; 95 % CI, 1.18–1.21) for each 1 % increase in RDW and by 44 % (OR 1.44; 95 % CI, 1.41–1.49) for each standard deviation (SD) increase in RDW. No effect modification was observed by the presence of anemia (P for interaction 0.45). This monotonic positive association and effect modification by anemia was similar for mean, peak, and nadir RDW (P < 0.0001 for all trends and P for interaction 0.55, 0.36, 0.43, respectively) (Fig. 1). Using the adjusted spline curves, the positive association between continuous RDW and all-cause mortality remained consistent, with the trend becoming slightly flatter after the inflection point (Fig. 2). The absolute risk difference in all-cause mortality also increased progressively among individuals in different RDW categories. The adjusted absolute risk difference for patients with baseline RDW ≥15.0 % was 40.0 (95 % CI, 29.6–52.0), indicating 40 additional deaths per 1000 patients compared with the reference group. Comparable results were obtained for the mean, peak and nadir RDW categories (Fig. 3).

### 3.3 Association between dynamic changes in RDW and all-cause mortality

**Fig. 4:**
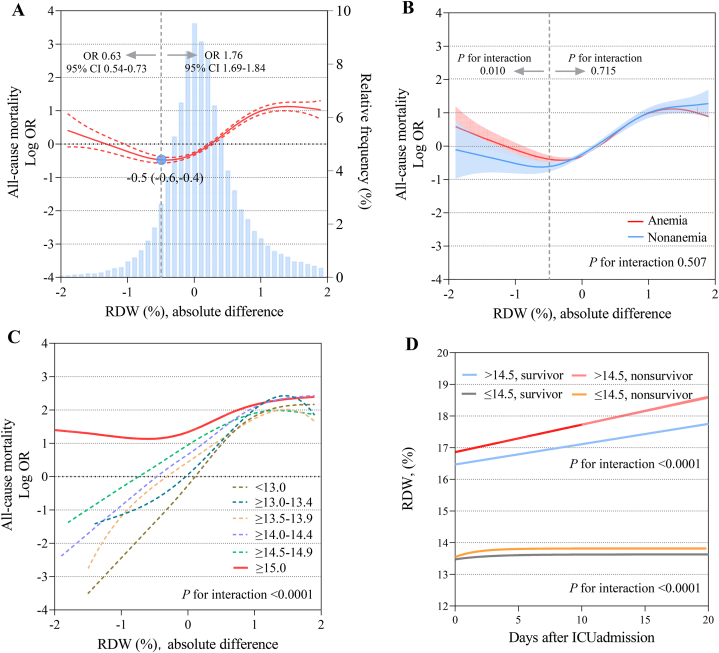
Association between changes in RDW and all-cause mortality Model was adjusted for all predefined covariates. (A) changes in RDW and all-cause mortality in overall cohort, (B) changes in RDW and all-cause mortality stratified by anemia, (C) changes in RDW and all-cause mortality stratified by RDW categories, and (D) time-varying trajectories of RDW. Absolute difference indicates the last RDW value minus the first RDW value during ICU stay. RDW, red blood cell distribution width. OR, odds ratio. CI, confidence interval.

The cubic spline curve showed a nonlinear relationship between changes in RDW and all-cause mortality. Patients with a 0.5 % (95 % CI 0.4–0.6 %) decline in RDW had the lowest risk of death, while declines beyond that appeared to be associated with a worse prognosis (OR 0.63, 95 % CI 0.54–0.73). In addition, an increase in RDW was associated with an increased risk of all-cause mortality (OR 1.76, 95 % CI 1.69–1.84) (Fig. 4-A). There was no significant effect alteration on the association between RDW changes and mortality by the presence of anemia (P for interaction 0.507). However, we tested the interaction of anemia separately before and after the inflection point (means 0.5 % decline) and found that there was an effect modification before the inflection point but not after (P for interaction of 0.010 and 0.715, respectively) (Fig. 4-B). Stratified analysis by RDW category showed no significant association between changes in RDW and mortality in patients with an RDW category ≥15.0 %. In other categories, a decrease in RDW was associated with a sustained reduction in the risk of death, whereas an increase in RDW was associated with an increased risk of death, followed by a plateau (Fig. 4-C). On the basis of these results, we reproduced the cubic spline curve between changes in RDW and mortality with the exclusion of individuals with RDW ≥15.0 %, showing that an increase in RDW was associated with an increase in mortality up to an inflection point of 1.0 %, and a declined in RDW was consistently associated with a decrease in mortality. The presence of anemia did not alter this association (eFig. 2 in the Supplement). The time-varying trajectories of RDW showed a mean RDW of 16.8 % in the elevated group compared with 13.5 % in the normal group. Patients who died had a consistently higher RDW than their surviving counterparts, regardless of whether the baseline RDW was normal or elevated (Fig. 4-D).

### 3.4 Association between RDW and cause-specific mortality

**Fig. 5:**
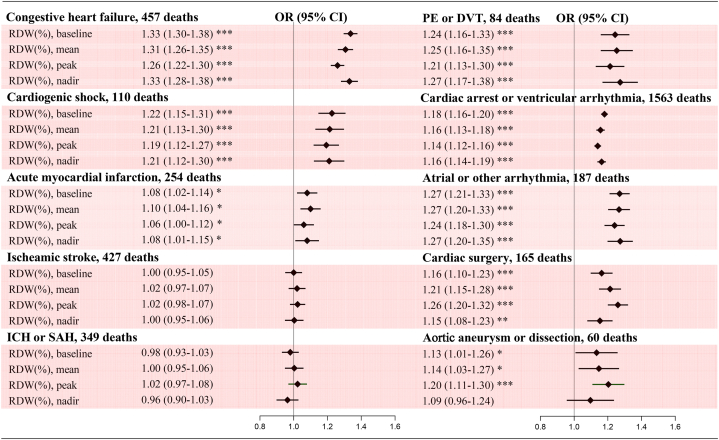
Adjusted odds ratios for cause-specific mortality by continuous RDW Model was adjusted for predefined covariates except primary disease on admission. ICH, intracerebral hemorrhage. SAH, subarachnoid hemorrhage. PE, pulmonary embolism. DVT, deep vein thrombosis. RDW, red blood cell distribution width. OR, odds ratio. CI, confidence interval.

The 10 cause-specific deaths accounted for 89.8 % of all deaths, with cardiac arrest or ventricular arrhythmia (38.4 %) and congestive heart failure (11.2 %) accounting for the largest proportions. There were positive associations between continuous RDW and most cause-specific deaths. Adjusted ORs showed strong associations of baseline, mean, peak and nadir RDW with congestive heart failure, cardiac arrest or ventricular arrhythmia, atrial or other arrhythmia, cardiogenic shock, cardiac surgery, and pulmonary embolism or deep vein thrombosis, but not with ischemic or hemorrhagic stroke. The associations of RDW with aortic aneurysm or dissection and acute myocardial infarction were weak, whereas the association of peak RDW with aortic aneurysm or dissection was strong (Fig. 5). There was also a significant risk gradient across categorical RDW, although the confidence intervals were wide because of the limited number of deaths for some conditions (eTable 2 in the Supplement).

### 3.5 Sensitivity analysis

In analyses stratified by age, sex, ethnicity, hematocrit ≥36 % or <36 %, MCV ≥80 fL or <80 fL, and mechanical ventilation, the risk of all-cause mortality was consistent with the overall cohort (eTable 3 in the Supplement). Excluding deaths occurring within 24 or 48 h of admission did not alter the results (eTable 4 and eTable 5 in the Supplement). Analysis of complete cases maintained similar results to the primary analysis (eTable 6 in the Supplement). Analysis using the actual mean, peak, and nadir RDW reconfirmed the association between RDW and all-cause mortality (eTable 7 in the Supplement). Cox proportional hazards model did not modify the results (eTable 8 in the Supplement). In the final multivariate model, RDW was the only laboratory variable with a statistically significant risk ratio for mortality (eTable 9 in the Supplement). Moreover, excluding deaths due to cardiogenic shock and cardiac arrest or ventricular arrhythmias, which had notably higher mortality rates, did not change the model framework much (eTable 10 in the Supplement).

## 4 Discussion

In this cohort study, we found a graded and independent association between RDW and all-cause mortality within 30 days of ICU admission. Patients with RDW ≥15.0 % exhibited a significantly increased mortality risk, with 40 additional deaths per 1000 patients compared to those with RDW <13.0 %. Notably, we extend previous findings that a high RDW, even within the range currently considered normal (13.5–14.5 %), is associated with an increased risk of mortality. These associations remained statistically significant after controlling for traditional risk factors and potential modifiers of anemia or transfusion status. A nonlinear relationship was observed between changes in RDW and all-cause mortality, varying by RDW category. Additionally, RDW showed a strong positive association with most, albeit not all, cause-specific deaths.

RDW has been proposed to be a pleiotropic biomarker of multidimensional physiological disturbance [18]. While a direct relationship has been established between RDW and the unfavorable outcomes of heart failure [19,20], myocardial infarction [21], atrial fibrillation [22], and other CVD [23], there is a paucity of data regarding the impact of RDW on clinical outcomes in ICU patients admitted for unselected cardiovascular reasons. In addition, previous studies linking RDW to adverse outcomes have been subject to several common limitations, including small sample sizes that result in inadequate statistical power, a focus on a narrow range of endpoints that limit outcome measures, the use of a single measure that fails to account for RDW fluctuations over time, and a simplistic classification of RDW as either normal or elevated that neglects a thorough examination of the shape of the association. In this study, we address these limitations as much as possible to provide robust evidence for a valuable biomarker.

Cardiovascular disease is one of the most common causes of ICU admission, accounting for one-third of adult admissions in the current study. We extracted and analyzed data for baseline, mean, peak, and nadir RDW separately. Four RDW metrics did not differ much, which is consistent with the well-established understanding that RDW represents the volume variance of a cell population and typically changes slowly (no more than 1 % or 2 % per day). Regardless of the metric employed, a stable positive dose-response relationship between RDW and all-cause mortality was observed. The cubic spline analysis also demonstrated a consistent trend, albeit with a slowdown at extremely high RDW levels. These findings are consistent with previous research in the context of general critical care conditions [14], reemphasize the feasibility of using this easily accessible hematologic parameter for risk stratification. In particular, we extend previous findings that high RDW even within the normal range (13.5–14.5 %) is associated with an increased risk of all-cause death, suggesting that RDW is a highly sensitive indicator and adding plausibility that mildly elevated RDW serves as an early warning sign of deteriorating health status. Differently, previous studies have shown that the RDW appears to be more strongly associated with total death in patients without anemia, with the possible explanation being that anemia itself is associated with greater anisocytosis, whereas the increased RDW in non-anemic patients may be primarily due to more pronounced disease severity and comorbidity burden other than anemia [12]. In the current study, we used four RDW measures and showed that the presence of anemia had no effect modification on the relationship between RDW and mortality. This may reflect the robust predictive value of RDW for adverse prognosis in the setting of cardiovascular critical illness, independent of anemia and other traditional risk factors. In this scenario, where disease severity and comorbidity burden are generally high across the cohort, anemia, a common contributor to RDW fluctuations, will likely no longer dominate the impact of RDW on mortality.

A nonlinear association between dynamic changes in RDW and overall mortality was found, characterized by a decline in mortality with a decrease in RDW up to the risk inflection point, and an increase in mortality with an increase in RDW, followed by a plateau. Further stratified analysis showed that, any decrease in RDW was associated with a significant reduction in mortality risk, whereas such a decrease did not confer a survival benefit in individuals with RDW ≥15.0 %. One possible explanation is that high RDW may serve as an indicator of disease severity rather than a direct pathological entity. The significantly increased RDW may signify a severe decompensation status, a malignant process that is arduous to reverse, or the overall advantages of correcting anisocytosis cannot be achieved in the short term. In the present study, patients with higher RDW levels were characterized by advanced age, higher APACHE scores, and a higher prevalence of cardiac arrest and cardiogenic shock. Of note, dynamic reductions in RDW were consistently associated with decreased mortality after excluding individuals with RDW ≥15.0 %. Due to the observational nature of the study, these findings are exploratory and necessitate a larger sample size to examine more refined classification and a longer follow-up period to assess the long-term effects of modifying RDW.

We further investigated the impact of RDW on death from 10 distinct cardiovascular disease phenotypes. RDW exhibited a strong positive association with death from most specific etiologies, including cardiac arrest or various arrhythmias, heart failure, cardiogenic shock, cardiac surgery, and pulmonary embolism or deep vein thrombosis. Moderate associations have also been shown for the less studied aortic aneurysm or dissection. Previous studies have revealed that in specific cohorts with myocardial infarction or stroke, RDW is a prognostic indicator in patients with these conditions [21,24]. However, we found a weak or zero association between RDW and death from myocardial infarction or stroke in this mixed CVD cohort. These findings suggest that the association pattern between RDW and cause-specific mortality varies between specific CVD cohorts and unselected critically ill CVD cohorts, highlighting the complex interactions of RDW with worsening health consequences in pooled populations with diverse clinical phenotypes, and thus urging efforts for a comprehensive and clear understanding of the biological pathways.

The biological mechanisms underlying the RDW-mortality association are not fully understood. Inflammatory responses and oxidative stress are proposed to be primarily associated with the imbalance in erythrocyte homeostasis [4]. Inflammatory cytokines impede erythrocyte maturation and induce cellular destruction or senescence, allowing the entry of juvenile reticulocytes into the peripheral circulation and delaying the elimination of ineffective cells, thereby distorting the RDW [25]. Reactive oxygen species can also decrease erythrocyte survival and increase the release of immature reticulocytes into the circulation [26]. In the setting of critical cardiovascular disease, the prevailing inflammatory response and oxidative stress lead to impaired iron metabolism, bone marrow hematopoiesis, and erythrocyte maturation, which in turn results to increased RDW. More characteristically, neurohumoral hyper-activation generates large amounts of deteriorating cardiac substrates including catecholamines and angiotensin, inducing a cascade of reactions including vasoconstriction, thrombosis, microcirculatory disturbances, and perfusion deficits, and resulting in corresponding tissue ischemia and hypoxia. Such alteration usually play negative effect on bone marrow and the hormone erythropoietin, which regulates the production, maturation, and survival of erythrocytes. These complex interactions make RDW a potential mediator of worsening disease status and poor prognosis. In this study, we demonstrated a powerful predictive value of RDW for mortality in a variety of cardiovascular disease phenotypes, including cardiac arrest, pulmonary embolism, and aortic aneurysm or dissection which have been less studied previously, suggesting that RDW may be a promising cardiovascular biomarker. Further studies are needed to explore whether its effect is independent of existing cardiovascular biomarkers such as brain natriuretic peptide and troponin.

The use of RDW as a prognosticator offers several advantages over other potential biomarkers. First, RDW appears to be a robust predictor that has been shown to improve conventional risk stratification [27] or to be incorporated into novel machine learning-based risk modeling frameworks [28]. Our findings lend further credence that RDW has a clinically significant and independent effect on adverse prognosis. Furthermore, the inclusion of an appropriate RDW category rather than a simplistic classification of normal or elevated may be a worthwhile option, as elevated RDW within the normal range was also associated with adverse outcomes. Second, RDW is readily available and inexpensive, and its accessibility and affordability allow it to be widely used in primary and advanced healthcare settings. Third, dynamically elevated RDW can help identify patients with rapid disease progression or significant decompensation and facilitate prompt and aggressive intervention. Correspondingly, dynamically reduced RDW may serve not only as a positive prognostic factor, but also as an indicator for evaluating treatment efficacy.

Several limitations should be noted. First, due to the inherent limitations of the observational design, we cannot establish causality. Second, there may still be residual confounders that would affect the results. We did not evaluate some potential confounders not available in the database (e.g., C-reactive protein, serum iron, folic acid, or vitamin B12 levels); therefore, we could not assess whether the effect of RDW was independent of these factors. Third, our cause-specific mortality analysis should be considered exploratory because the relative paucity of events limits statistical power and prevents us from providing specific insights into mechanisms. Fourth, the lack of long-term follow-up results is also an important limitation, and since this information is not available in the database. Finally, it should be noted that this study included only cardiovascular patients treated in the ICU, and the findings cannot be extended to other cardiovascular patients in cardiology or outpatient settings.

## 5 Conclusion

RDW is a strong predictor of all-cause and cause-specific mortality in critically ill cardiovascular patients. The current application of this readily available parameter has been grossly neglected. Our findings highlight the importance of using this ubiquitous metric for rapid and dynamic risk assessment.

## Ethics approval and consent to participate

The Massachusetts Institute of Technology approved the use of the database, and the requirement for patient consent and an ethical approval statement was waived because of the retrospective design, lack of direct patient intervention, and a security system certified by an independent privacy expert (Privacert, Cambridge, MA) as meeting Safe Harbor standards for risk of re-identification (Health Insurance Portability and Accountability Act Certification No. 1031219-2).

## Funding/support

None.

## Data availability

Data associated with this study have been deposited into a publicly available repository, available at https://www. physionet. org/content/eicu-crd-demo/2.0.1/.

## CRediT authorship contribution statement

**Shan Li:** Conceptualization, Formal analysis, Methodology, Project administration, Supervision, Writing – review & editing. **Wei Zhang:** Data curation, Formal analysis, Investigation, Validation, Writing – original draft. **Xiao Liang:** Data curation, Formal analysis, Resources, Software, Writing – original draft.

## Declaration of competing interest

The authors declare no potential conflict of interest.
