# Supplementary material for: Red blood cell distribution width and mortality risk in critically ill cardiovascular patients
Source: Heliyon. 2023 Nov 11;9(11):e22225. doi: 10.1016/j.heliyon.2023.e22225 (PMC10692801; doi:10.1016/j.heliyon.2023.e22225)
Supplement: Multimedia component 1 [file mmc1.docx]

**Supplementary material**

**Red Blood Cell Distribution Width and Mortality Risk**

**in Critically Ill Cardiovascular Patients**

Shan Li, Wei Zhang, Xiao Liang

**Legends**

**eFigure 1. The mean RDW values for baseline, mean, peak and nadir measurements**

**eFigure 2. Association between changes in RDW and all-cause mortality with excluding individuals with RDW > 15%**

**eTable 1. Baseline characteristics of individuals included or not included in the analysis**

**eTable 2. Univariable and multivariable adjusted odds ratios for cause-specific mortality by categorical RDW**

**eTable 3. Stratified analyses for all-cause mortality by categorical RDW**

**eTable 4. Univariable and multivariable adjusted odds ratios for all-cause mortality by categorical RDW after excluding patients died within 24 hours of admission**

**eTable 5. Univariable and multivariable adjusted odds ratios for all-cause mortality by categorical RDW after excluding patients died within 48 hours of admission**

**eTable 6. Univariable and multivariable adjusted odds ratios for all-cause mortality by categorical RDW with complete case analysis**

**eTable 7. Univariable and multivariable adjusted odds ratios for all-cause mortality by categorical RDW in patients with multiple measures**

**eTable 8. Univariable and multivariable adjusted odds ratios for all-cause mortality by categorical RDW with cox proportional hazards model**

**eTable 9. Multivariable logistic regression model for all-cause mortality**

**eTable 10. Multivariable logistic regression model for all-cause mortality after excluding cardiac arrest or ventricular arrhythmia and cardiogenic shock**

**eFigure 1.** The mean RDW values for baseline, mean, peak and nadir measurements


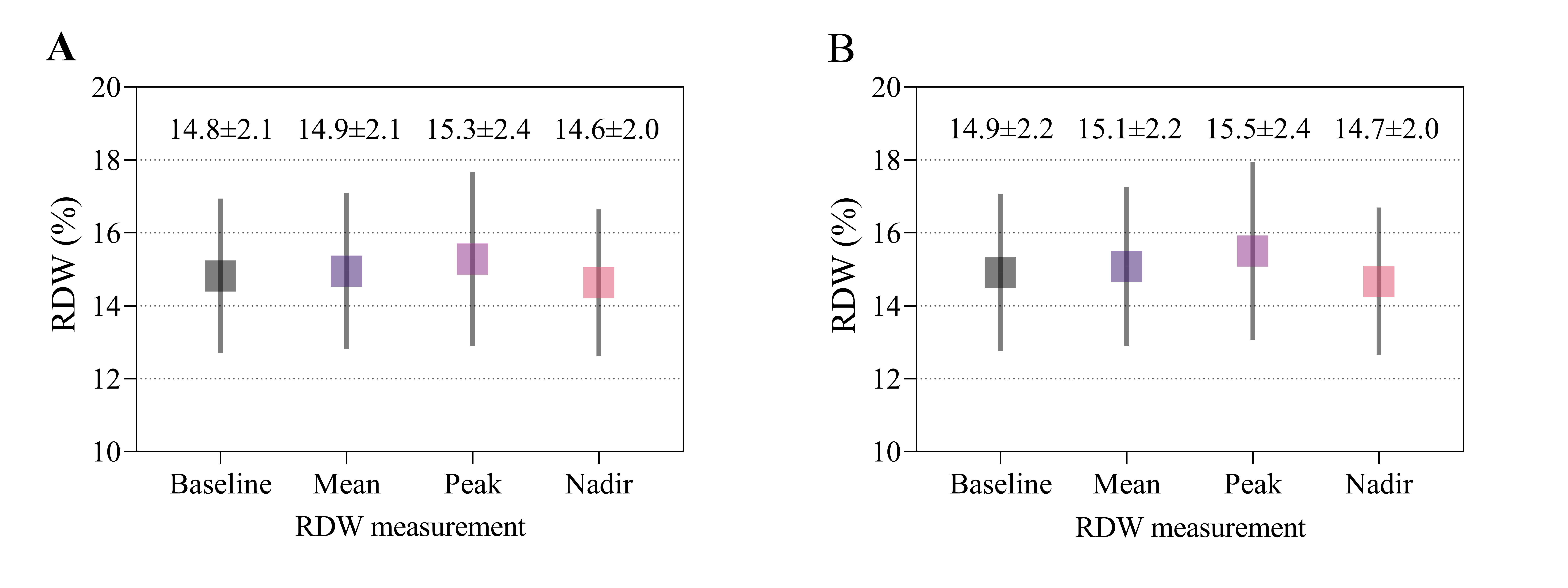


1. Entire cohort, n=47266, (B) Patients with multiple measurements, n=36723.

**eFigure 2.** Association between changes in RDW and all-cause mortality with excluding individuals with RDW > 15%


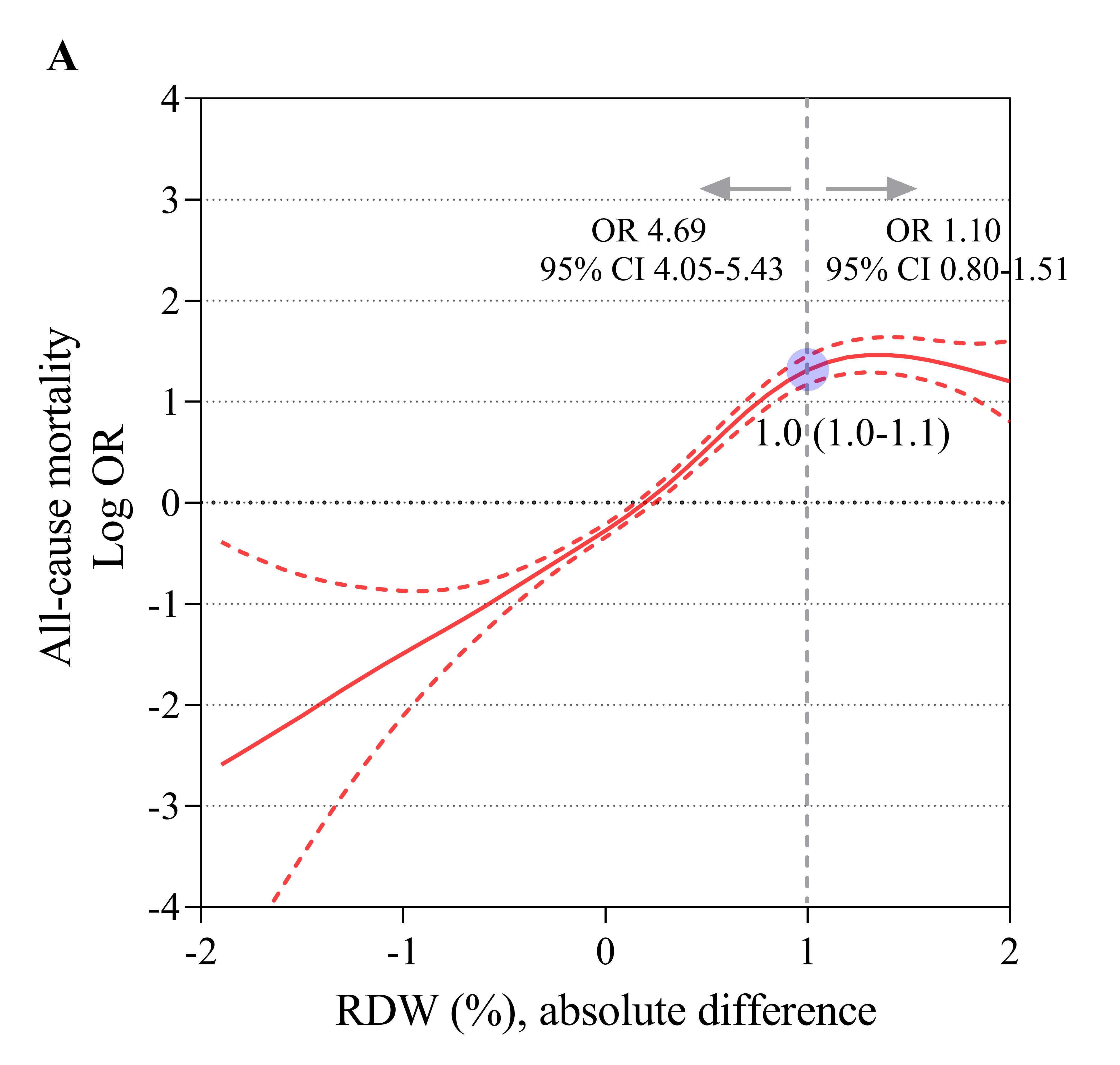

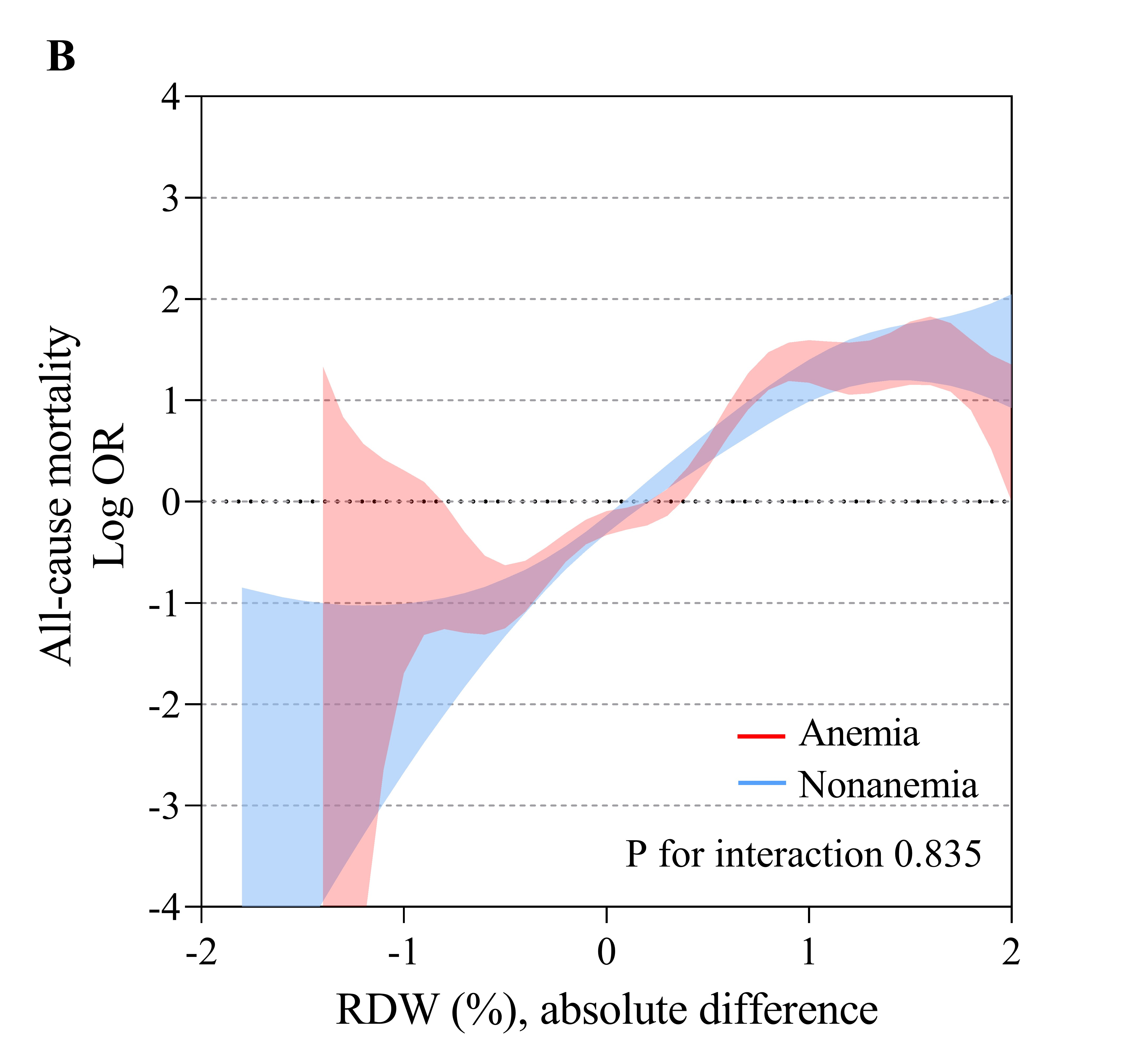


Model was adjusted for all predefined covariates. (A) changes in RDW and all-cause mortality in overall cohort, (B) changes in RDW and all-cause mortality stratified by anemia. Absolute difference indicates the last RDW value minus the first RDW value during ICU stay. RDW, red blood cell distribution width. OR, odds ratio. CI, confidence interval.

**eTable 1.** Baseline characteristics of individuals included or not included in the analysis

|  | Not included in analysis | Included in analysis | Absolute standardized difference |
| --- | --- | --- | --- |
| N (%) | 14403 (23.4) | 47266 (76.6) |  |
| Age, years | 65.9 ±15.0 | 66.9 ±14.5 | 0.069 |
| Male, n (%) | 8180 (56.8) | 27619 (58.4) | 0.033 |
| Caucasian, n (%) | 10636 (73.8) | 36846 (78.0) | 0.096 |
| Body mass index, kg/m^2^ | 29.2 ±7.1 | 29.3 ±7.1 | 0.007 |
| Severity score |  |  |  |
| Glasgow coma score | 13 ±4 | 13 ±4 | 0.011 |
| APACHE score | 52 ±27 | 55 ±26 | 0.110 |
| Heart rate, bpm | 93 ±34 | 94 ±32 | 0.057 |
| Mean blood pressure, mmHg | 92 ±41 | 92 ±43 | 0.048 |
| Laboratory measurement |  |  |  |
| White blood cell, ×10^9^/L | 12.1 ±7.4 | 11.7 ±6.9 | 0.052 |
| Hemoglobin, g/dL | 11.5 ±2.2 | 11.6 ±2.2 | 0.011 |
| Hematocrit, % | 34.2±6.9 | 33.9±6.7 | 0.059 |
| Mean corpuscular volume, fL | 89.8±6.5 | 90.0±6.2 | 0.072 |
| Albumin, g/L | 31.1 ±6.6 | 31.0 ±6.4 | 0.015 |
| Creatinine, mg/dL | 1.1 (0.4-12.7) | 1.0 (0.5-13.0) | 0.082 |
| Primary diagnosis |  |  |  |
| Ischemic stroke, n (%) | 1677 (11.6) | 4614 (9.8) | 0.061 |
| Intracerebral hemorrhage or [subarachnoid hemorrhage](https://www.findacode.com/code-set.php?set=ICD9&i=1023) , n (%) | 1043 (7.2) | 2401 (5.1) | 0.071 |
| Acute myocardial infarction, n (%) | 1789 (12.4) | 5634 (11.9) | 0.015 |
| Congestive heart failure, n (%) | 1241 (8.6) | 4767 (10.1) | 0.050 |
| Cardiac arrest or ventricular arrhythmia, n(%) | 1462 (10.2) | 4065 (8.6) | 0.053 |
| Atrial or other arrhythmia, n (%) | 1118 (7.8) | 3243 (6.9) | 0.035 |
| Hypertension, n (%) | 568 (3.9) | 1356 (2.9) | 0.059 |
| Cardiac surgery, n (%) | 1271 (8.8) | 8532 (18.1) | 0.273 |
| Cardiogenic shock, n (%) | 90 (0.6) | 424 (0.9) | 0.031 |
| Cardiomyopathy, n (%) | 104 (0.7) | 421 (0.9) | 0.019 |
| Pulmonary embolism or deep venous thrombosis, n (%) | 400 (2.8) | 1695 (3.6) | 0.046 |
| Aortic aneurysm or dissection, n (%) | 323 (2.2) | 1107 (2.3) | 0.007 |
| Pre-admission comorbidities |  |  |  |
| Hypertension, n (%) | 5035 (35.0) | 27715 (58.6) | 0.489 |
| Stroke or TIA, n (%) | 1360 (9.4) | 5713 (12.1) | 0.074 |
| Prior myocardial infarction, n (%) | 1257 (8.7) | 6448 (13.6) | 0.156 |
| PCI, n (%) | 966 (6.7) | 4887 (10.3) | 0.130 |
| CABG, n (%) | 804 (5.6) | 3710 (7.8) | 0.041 |
| Chronic heart failure, n (%) | 1999 (13.9) | 9334 (19.7) | 0.090 |
| Atrial fibrillation, n (%) | 1387 (9.6) | 6646 (14.1) | 0.086 |
| Diabetes mellitus, n (%) | 2012 (14.0) | 6102 (12.9) | 0.027 |
| COPD, n (%) | 1417 (9.8) | 5950 (12.6) | 0.077 |
| Renal insufficiency, n (%) | 1463 (10.2) | 6458 (13.7) | 0.042 |
| Dementia, n (%) | 251 (1.7) | 1150 (2.4) | 0.048 |
| Cancer, n (%) | 1365 (9.5) | 5434 (11.5) | 0.041 |
| Theraputics |  |  |  |
| Mechanical ventilation, n (%) | 2985 (20.7) | 11598 (24.5) | 0.089 |
| Dialysis, n (%) | 511 (3.5) | 1825 (3.9) | 0.016 |
| Red blood cell transfusion, n (%) | 183 (1.3) | 1061 (2.2) | 0.074 |
| Anticoagulant, n (%) | 2790 (19.4) | 10532 (22.3) | 0.062 |
| Antiplatelet, n (%) | 3456 (24.0) | 13002 (27.5) | 0.070 |
| Lipid-lowering, n (%) | 4335 (30.1) | 15807 (33.4) | 0.035 |

Given the large sample size, we calculated absolute standardized differences (ASDs) to compare patients with RDW measurement who were included in the analysis with those without RDW measurement who were not included in the analysis. RDW, red blood cell distribution width. APACHE, acute physiology, age and chronic health evaluation.

**eTable 2.** Univariable and multivariable adjusted odds ratios for cause-specific mortality by categorical RDW

| RDW (%) category | <13.0 | 13.0-13.4 | 13.5-13.9 | 14.0-14.4 | ≥14.5 |
| --- | --- | --- | --- | --- | --- |
|  | Reference | OR (95% CI) | OR (95% CI) | OR (95% CI) | OR (95% CI) |
| **Cardiac arrest or ventricular arrhythmia** | | | | | |
| Model I | 1 | 1.03 (0.80, 1.32) | 1.11 (0.87, 1.42) | 1.28 (1.00, 1.64) | 2.52 (2.06, 3.07) |
| Model II | 1 | 1.07 (0.83, 1.37) | 1.18 (0.92, 1.52) | 1.39 (1.08, 1.79) | 2.75 (2.25, 3.36) |
| Model III | 1 | 1.07 (0.83, 1.39) | 1.19 (0.93, 1.53) | 1.40 (1.09, 1.81) | 2.71 (2.20, 3.34) |
| **Atrial or other arrhythmia** | | | | | |
| Model I | 1 | 0.82 (0.17, 4.06) | 4.53 (1.33, 15.45) | 4.62 (1.34, 15.98) | 13.31 (4.24, 41.75) |
| Model II | 1 | 0.71 (0.14, 3.54) | 3.39 (0.98, 11.74) | 3.55 (1.04, 12.13) | 9.65 (3.07, 30.34) |
| Model III | 1 | 0.75 (0.15, 3.71) | 3.63 (1.04, 12.68) | 3.76 (1.09, 12.94) | 10.16 (3.19, 32.30) |
| **Cardiogenic shock** | | | | | |
| Model I | 1 | 1.50 (0.55, 4.06) | 0.93 (0.31, 2.77) | 0.92 (0.30, 2.86) | 3.56 (1.55, 8.16) |
| Model II | 1 | 1.45 (0.54, 3.94) | 0.88 (0.29, 2.62) | 0.86 (0.28, 2.68) | 3.31 (1.43, 7.64) |
| Model III | 1 | 1.48 (0.54, 4.04) | 0.90 (0.30, 2.72) | 0.87 (0.28, 2.75) | 3.48 (1.47, 8.27) |
| **Congestive heart failure** | | | | | |
| Model I | 1 | 5.33 (1.20, 23.62) | 7.59 (1.77, 32.60) | 14.84 (3.55, 61.92) | 53.01 (13.21, 90.72) |
| Model II | 1 | 4.85 (1.09, 21.50) | 6.44 (1.50, 27.66) | 12.06 (2.89, 50.39) | 42.78 (10.65, 82.85) |
| Model III | 1 | 4.67 (1.05, 20.74) | 5.94 (1.38, 25.58) | 10.99 (2.62, 46.04) | 32.52 (9.06, 76.23) |
| **Cardiac surgery** | | | | | |
| Model I | 1 | 1.91 (0.49, 7.39) | 4.53 (1.33, 15.45) | 4.93 (1.44, 16.93) | 10.88 (3.46, 34.23) |
| Model II | 1 | 1.86 (0.48, 7.21) | 4.34 (1.27, 14.84) | 4.65 (1.35, 16.02) | 10.25 (3.25, 32.37) |
| Model III | 1 | 1.87 (0.48, 7.24) | 4.24 (1.23, 14.59) | 4.50 (1.30, 15.64) | 9.71 (3.04, 31.02) |
| **Pulmonary embolism or deep venous thrombosis** | | | | | |
| Model I | 1 | 3.28 (0.37, 29.31) | 4.79 (0.58, 39.77) | 10.17 (1.31, 68.77) | 16.55 (2.29, 80.40) |
| Model II | 1 | 3.20 (0.36, 28.65) | 4.59 (0.55, 38.21) | 9.51 (1.22, 63.94) | 15.43 (2.13, 80.79) |
| Model III | 1 | 3.35 (0.37, 30.13) | 4.85 (0.58, 40.72) | 10.22 (1.30, 60.21) | 15.92 (2.17, 80.80) |
| **Acute myocardial infarction** | | | | | |
| Model I | 1 | 1.32 (0.73, 2.38) | 1.60 (0.91, 2.82) | 1.80 (1.02, 3.18) | 2.02 (1.23, 3.30) |
| Model II | 1 | 1.18 (0.65, 2.13) | 1.31 (0.74, 2.32) | 1.40 (0.79, 2.49) | 1.56 (0.95, 2.56) |
| Model III | 1 | 1.18 (0.65, 2.14) | 1.34 (0.75, 2.39) | 1.44 (0.80, 2.59) | 1.78 (1.02, 2.65) |
| **Ischemic stroke** | | | | | |
| Model I | 1 | 1.25 (0.83, 1.89) | 1.47 (0.99, 2.19) | 1.49 (0.99, 2.23) | 1.41 (0.99, 1.99) |
| Model II | 1 | 1.15 (0.76, 1.74) | 1.27 (0.85, 1.89) | 1.22 (0.81, 1.84) | 1.13 (0.80, 1.61) |
| Model III | 1 | 1.15 (0.76, 1.74) | 1.28 (0.85, 1.92) | 1.24 (0.82, 1.89) | 1.14 (0.79, 1.65) |
| **Intracerebral hemorrhage or subarachnoid hemorrhage** | | | | | |
| Model I | 1 | 1.05 (0.72, 1.53) | 0.65 (0.43, 0.99) | 0.94 (0.63, 1.40) | 0.80 (0.58, 1.10) |
| Model II | 1 | 1.07 (0.73, 1.55) | 0.65 (0.43, 1.00) | 0.94 (0.63, 1.39) | 0.78 (0.56, 1.08) |
| Model III | 1 | 1.09 (0.74, 1.59) | 0.68 (0.45, 1.05) | 0.98 (0.65, 1.48) | 0.84 (0.59, 1.19) |

Model I was unadjusted. Model II was adjusted for age, sex and ethnicity. Model III was adjusted for age, sex, ethnicity, BMI, heart rate, mean blood pressure, GCS, APACHE score, white blood cell count, hemoglobin, hematocrit, mean corpuscular volume, albumin, creatinine, prior comorbidities (myocardial infarction, percutaneous coronary intervention, coronary artery bypass grafting, chronic heart failure, atrial fibrillation, diabetes mellitus, hypertension, stroke or transient ischemic attack, chronic obstructive pulmonary disease, renal insufficiency, dementia, and cancer), and treatments (mechanical ventilation, dialysis, red blood cell transfusion, anticoagulants, antiplatelet agents, and lipid-lowering agents). The risk estimates for aortic aneurysm or dissection were not shown, and the confidence intervals were too wide due to the limited number of events.

**eTable 3.** Stratified analyses for all-cause mortality by categorical RDW

| RDW (%) category | <13.0 | 13.0-13.4 | 13.5-13.9 | 14.0-14.4 | 14.5-14.9 | ≥15.0 | *P* for interaction |
| --- | --- | --- | --- | --- | --- | --- | --- |
|  | Reference | OR (95% CI) | OR (95% CI) | OR (95% CI) | OR (95% CI) | OR (95% CI) |  |
| **Baseline RDW** | | | | | | | |
| **Age** | | | | | | | 0.448 |
| <70 years | 1 | 1.06 (0.84, 1.32) | 1.14 (0.91, 1.43) | 1.43 (1.13, 1.81) | 1.51 (1.18, 1.92) | 2.75 (2.27, 3.33) |  |
| ≥70 years | 1 | 1.46 (1.10, 1.96) | 1.65 (1.25, 2.18) | 2.04 (1.55, 2.70) | 2.60 (1.97, 3.43) | 3.57 (2.77, 4.60) |  |
| **Sex** | | | | | | | 0.118 |
| Male | 1 | 1.16 (0.92, 1.45) | 1.32 (1.05, 1.65) | 1.64 (1.30, 2.06) | 1.87 (1.48, 2.36) | 3.06 (2.51, 3.73) |  |
| Female | 1 | 1.16 (0.89, 1.53) | 1.24 (0.95, 1.62) | 1.51 (1.15, 1.96) | 1.85 (1.41, 2.42) | 2.65 (2.10, 3.34) |  |
| **Ethnicity** | | | | | | | 0.173 |
| Caucasians | 1 | 1.11 (0.92, 1.35) | 1.32 (1.09, 1.59) | 1.64 (1.35, 1.98) | 2.07 (1.71, 2.52) | 2.98 (2.52, 3.52) |  |
| Others | 1 | 1.42 (0.95, 2.11) | 1.36 (0.98, 1.75) | 1.56 (1.02, 2.18) | 1.74 (1.10, 2.42) | 2.67 (1.90, 3.75) |  |
| **Hematocrit** | | | | | | | 0.561 |
| <0.36 | 1 | 1.19 (0.96, 1.47) | 1.32 (1.07, 1.64) | 1.60 (1.29, 2.00) | 2.11 (1.69, 2.64) | 3.09 (2.58, 3.69) |  |
| ≥0.36 | 1 | 1.07 (0.78, 1.47) | 1.18 (0.88, 1.60) | 1.48 (1.10, 1.99) | 1.58 (1.17, 2.14) | 2.52 (1.91, 3.32) |  |
| **MCV** | | | | | | | 0.388 |
| <80 | 1 | 0.84 (0.59, 1.19) | 1.10 (0.79, 1.53) | 1.39 (1.02, 1.79) | 1.50 (1.07, 2.11) | 2.25 (1.69, 2.99) |  |
| ≥80 | 1 | 1.30 (1.06, 1.59) | 1.37 (1.12, 1.67) | 1.73 (1.41, 2.12) | 2.06 (1.68, 2.53) | 3.19 (2.68, 3.81) |  |
| **Mechanical ventilation** | | | | | | | 0.228 |
| Yes | 1 | 1.14 (0.92, 1.41) | 1.25 (1.02, 1.54) | 1.52 (1.24, 1.87) | 1.79 (1.45, 2.20) | 2.74 (2.29, 3.29) |  |
| No | 1 | 1.16 (0.85, 1.58) | 1.31 (0.95, 1.81) | 1.72 (1.24, 2.37) | 2.17 (1.55, 3.05) | 3.43 (2.62, 4.48) |  |
| **Mean RDW** | | | | | | | |
| **Age** | | | | | | | 0.452 |
| <70 years | 1 | 1.30 (1.00, 1.70) | 1.94 (1.50, 2.51) | 2.00 (1.53, 2.60) | 3.03 (2.33, 3.94) | 4.73 (3.78, 5.93) |  |
| ≥70 years | 1 | 1.86 (1.31, 2.62) | 2.19 (1.57, 3.06) | 2.94 (2.11, 4.08) | 3.83 (2.76, 5.32) | 5.39 (3.96, 7.33) |  |
| **Sex** | | | | | | | 0.285 |
| Male | 1 | 1.52 (1.16, 1.98) | 1.92 (1.48, 2.48) | 2.32 (1.79, 3.00) | 3.24 (2.50, 4.19) | 4.94 (3.92, 6.22) |  |
| Female | 1 | 1.39 (1.00, 1.95) | 1.99 (1.45, 2.73) | 2.28 (1.66, 3.13) | 3.18 (2.32, 4.35) | 4.41 (3.31, 5.87) |  |
| **Ethnicity** | | | | | | | 0.584 |
| Caucasians | 1 | 1.42 (1.13, 1.78) | 1.90 (1.53, 2.36) | 2.33 (1.87, 2.90) | 3.30 (2.65, 4.11) | 4.75 (3.90, 5.78) |  |
| Others | 1 | 1.77 (1.06, 2.95) | 2.35 (1.45, 3.82) | 2.39 (1.47, 3.89) | 3.28 (2.03, 5.30) | 5.03 (3.26, 7.77) |  |
| **Hematocrit** | | | | | | | 0.965 |
| <0.36 | 1 | 1.46 (1.14, 1.87) | 2.03 (1.59, 2.58) | 2.27 (1.78, 2.91) | 3.40 (2.66, 4.34) | 4.84 (3.93, 5.98) |  |
| ≥0.36 | 1 | 1.49 (1.01, 2.21) | 1.85 (1.27, 2.70) | 2.36 (1.63, 3.42) | 3.09 (2.13, 4.47) | 4.60 (3.24, 6.52) |  |
| **MCV** | | | | | | | 0.367 |
| <80 | 1 | 1.15 (0.76, 1.73) | 1.61 (1.10, 2.36) | 1.66 (1.13, 2.45) | 2.81 (1.92, 4.12) | 3.59 (2.56, 5.05) |  |
| ≥80 | 1 | 1.60 (1.26, 2.04) | 2.09 (1.65, 2.64) | 2.62 (2.07, 3.31) | 3.44 (2.72, 4.35) | 5.24 (4.24, 6.46) |  |
| **Mechanical ventilation** | | | | | | | 0.248 |
| Yes | 1 | 1.47 (1.14, 1.89) | 1.88 (1.48, 2.40) | 2.25 (1.77, 2.86) | 3.19 (2.51, 4.06) | 4.52 (3.63, 5.63) |  |
| No | 1 | 1.45 (1.01, 2.10) | 2.12 (1.48, 3.03) | 2.46 (1.71, 3.55) | 3.31 (2.28, 4.80) | 5.61 (4.10, 7.67) |  |
| **Peak RDW** | | | | | | | |
| **Age** | | | | | | | 0.118 |
| <70 years | 1 | 1.15 (0.83, 1.59) | 1.60 (1.17, 2.18) | 2.14 (1.57, 2.92) | 2.68 (1.97, 3.63) | 4.93 (3.76, 6.45) |  |
| ≥70 years | 1 | 1.65 (1.09, 2.51) | 2.42 (1.64, 3.58) | 2.53 (1.71, 3.74) | 3.79 (2.57, 5.57) | 5.59 (3.87, 8.06) |  |
| **Sex** | | | | | | | 0.420 |
| Male | 1 | 1.24 (0.90, 1.71) | 1.82 (1.34, 2.46) | 2.20 (1.63, 2.98) | 2.88 (2.14, 3.89) | 4.98 (3.79, 6.54) |  |
| Female | 1 | 1.43 (0.95, 2.17) | 2.05 (1.39, 3.02) | 2.25 (1.53, 3.30) | 3.30 (2.26, 4.82) | 4.98 (3.50, 7.09) |  |
| **Ethnicity** | | | | | | | 0.933 |
| Caucasians | 1 | 1.24 (0.94, 1.63) | 1.81 (1.40, 2.34) | 2.20 (1.70, 2.84) | 3.06 (2.37, 3.95) | 4.87 (3.86, 6.15) |  |
| Others | 1 | 1.72 (0.89, 3.33) | 2.52 (1.36, 4.66) | 2.51 (1.35, 4.67) | 3.47 (1.90, 6.34) | 6.00 (3.42, 10.53) |  |
| **Hematocrit** | | | | | | | 0.781 |
| <0.36 | 1 | 1.19 (0.88, 1.61) | 1.96 (1.48, 2.59) | 2.18 (1.64, 2.89) | 3.01 (2.28, 3.99) | 5.05 (3.95, 6.46) |  |
| ≥0.36 | 1 | 1.58 (0.96, 2.62) | 1.89 (1.17, 3.06) | 2.35 (1.46, 3.77) | 3.25 (2.03, 5.20) | 5.08 (3.24, 7.99) |  |
| **MCV** | | | | | | | 0.245 |
| <80 | 1 | 0.80 (0.48, 1.33) | 1.63 (1.05, 2.54) | 1.79 (1.15, 2.79) | 2.04 (1.30, 3.19) | 3.55 (2.37, 5.31) |  |
| ≥80 | 1 | 1.54 (1.15, 2.07) | 2.00 (1.51, 2.65) | 2.40 (1.81, 3.18) | 3.57 (2.71, 4.70) | 5.64 (4.37, 7.28) |  |
| **Mechanical ventilation** | | | | | | | 0.291 |
| Yes | 1 | 1.38 (1.01, 1.88) | 1.84 (1.37, 2.46) | 2.15 (1.61, 2.88) | 3.13 (2.35, 4.16) | 4.81 (3.68, 6.28) |  |
| No | 1 | 1.13 (0.73, 1.76) | 2.04 (1.35, 3.09) | 2.38 (1.57, 3.59) | 2.67 (1.73, 4.13) | 5.70 (3.96, 8.21) |  |
| **Nadir RDW** | | | | | | | |
| **Age** | | | | | | | 0.962 |
| <70 years | 1 | 1.19 (0.96, 1.48) | 1.45 (1.16, 1.80) | 1.86 (1.48, 2.33) | 1.93 (1.52, 2.45) | 3.43 (2.86, 4.12) |  |
| ≥70 years | 1 | 1.82 (1.39, 2.39) | 1.89 (1.45, 2.47) | 2.63 (2.02, 3.41) | 3.33 (2.55, 4.33) | 4.17 (3.28, 5.30) |  |
| **Sex** | | | | | | | 0.060 |
| Male | 1 | 1.43 (1.15, 1.77) | 1.57 (1.27, 1.95) | 2.04 (1.64, 2.54) | 2.60 (2.08, 3.25) | 3.76 (3.12, 4.54) |  |
| Female | 1 | 1.30 (1.00, 1.68) | 1.48 (1.15, 1.91) | 1.99 (1.55, 2.56) | 2.15 (1.66, 2.79) | 3.09 (2.47, 3.85) |  |
| **Ethnicity** | | | | | | | 0.276 |
| Caucasians | 1 | 1.36 (1.14, 1.64) | 1.57 (1.31, 1.88) | 2.13 (1.78, 2.56) | 2.66 (2.21, 3.20) | 3.59 (3.06, 4.21) |  |
| Others | 1 | 1.45 (0.99, 2.12) | 1.41 (0.97, 2.06) | 1.83 (1.26, 2.67) | 1.74 (1.18, 2.57) | 3.17 (2.30, 4.37) |  |
| **Hematocrit** | | | | | | | 0.822 |
| <0.36 | 1 | 1.48 (1.21, 1.81) | 1.54 (1.25, 1.89) | 2.05 (1.66, 2.54) | 2.61 (2.10, 3.25) | 3.65 (3.07, 4.34) |  |
| ≥0.36 | 1 | 1.21 (0.91, 1.62) | 1.49 (1.13, 1.97) | 1.98 (1.50, 2.62) | 2.18 (1.65, 2.89) | 3.19 (2.47, 4.11) |  |
| **MCV** | | | | | | | 0.529 |
| <80 | 1 | 1.05 (0.75, 1.46) | 1.42 (1.04, 1.94) | 1.69 (1.23, 2.32) | 2.10 (1.52, 2.92) | 2.86 (2.18, 3.76) |  |
| ≥80 | 1 | 1.50 (1.24, 1.82) | 1.59 (1.31, 1.92) | 2.20 (1.82, 2.67) | 2.57 (2.11, 3.12) | 3.74 (3.16, 4.41) |  |
| **Mechanical ventilation** | | | | | | | 0.367 |
| Yes | 1 | 1.32 (1.08, 1.61) | 1.49 (1.23, 1.81) | 1.97 (1.63, 2.40) | 2.30 (1.89, 2.81) | 3.32 (2.80, 3.95) |  |
| No | 1 | 1.47 (1.09, 1.98) | 1.60 (1.17, 2.18) | 2.18 (1.59, 3.00) | 2.79 (2.02, 3.86) | 4.01 (3.10, 5.20) |  |

Model was adjusted for all predefined covariates except stratified variables.

**eTable 4.** Univariable and multivariable adjusted odds ratios for all-cause mortality by categorical RDW after excluding patients died within 24 hours of admission (n=46560)

| RDW (%) category | <13.0 | 13.0-13.4 | 13.5-13.9 | 14.0-14.4 | 14.5-14.9 | ≥15.0 | *P* for trend |
| --- | --- | --- | --- | --- | --- | --- | --- |
|  | Reference | OR (95% CI) | OR (95% CI) | OR (95% CI) | OR (95% CI) | OR (95% CI) |  |
| **Baseline RDW** | | | | | | | |
| Model I | 1 | 1.16 (0.98, 1.38) | 1.33 (1.13, 1.56) | 1.63 (1.39, 1.92) | 2.04 (1.73, 2.40) | 3.33 (2.91, 3.82) |  |
| Model II | 1 | 1.13 (0.96, 1.34) | 1.27 (1.08, 1.50) | 1.54 (1.31, 1.81) | 1.91 (1.62, 2.25) | 3.15 (2.74, 3.61) |  |
| Model III | 1 | 1.15 (0.97, 1.38) | 1.27 (1.07, 1.51) | 1.58 (1.33, 1.88) | 1.88 (1.57, 2.24) | 2.87 (2.47, 3.34) | <0.0001 |
| **Mean RDW** | | | | | | | |
| Model I | 1 | 1.44 (1.18, 1.77) | 1.87 (1.55, 2.27) | 2.30 (1.90, 2.79) | 3.26 (2.70, 3.94) | 5.04 (4.26, 5.97) |  |
| Model II | 1 | 1.41 (1.15, 1.73) | 1.80 (1.48, 2.18) | 2.19 (1.81, 2.66) | 3.09 (2.55, 3.74) | 4.79 (4.04, 5.68) |  |
| Model III | 1 | 1.48 (1.20, 1.82) | 1.94 (1.58, 2.37) | 2.33 (1.91, 2.86) | 3.26 (2.66, 3.99) | 4.75 (3.96, 5.70) | <0.0001 |
| **Peak RDW** | | | | | | | |
| Model I | 1 | 1.33 (1.04, 1.71) | 1.89 (1.50, 2.39) | 2.31 (1.83, 2.91) | 3.29 (2.62, 4.13) | 5.56 (4.52, 6.84) |  |
| Model II | 1 | 1.31 (1.02, 1.68) | 1.82 (1.44, 2.31) | 2.20 (1.74, 2.77) | 3.12 (2.49, 3.93) | 5.28 (4.29, 6.51) |  |
| Model III | 1 | 1.33 (1.03, 1.72) | 1.88 (1.48, 2.40) | 2.24 (1.76, 2.85) | 3.09 (2.43, 3.92) | 5.02 (4.03, 6.25) | <0.0001 |
| **Nadir RDW** | | | | | | | |
| Model I | 1 | 1.30 (1.11, 1.52) | 1.48 (1.27, 1.73) | 1.92 (1.64, 2.24) | 2.42 (2.07, 2.82) | 3.74 (3.29, 4.26) |  |
| Model II | 1 | 1.26 (1.07, 1.47) | 1.41 (1.21, 1.65) | 1.81 (1.55, 2.12) | 2.27 (1.94, 2.65) | 3.55 (3.11, 4.04) |  |
| Model III | 1 | 1.36 (1.15, 1.60) | 1.52 (1.29, 1.79) | 2.03 (1.72, 2.40) | 2.41 (2.04, 2.86) | 3.45 (2.99, 3.98) | <0.0001 |

Model I was unadjusted. Model II was adjusted for age, sex and ethnicity. Model III was adjusted for all predefined covariates.

**eTable 5.** Univariable and multivariable adjusted odds ratios for all-cause mortality by categorical RDW after excluding patients died within 48 hours of admission (n=45915)

| RDW (%) category | <13.0 | 13.0-13.4 | 13.5-13.9 | 14.0-14.4 | 14.5-14.9 | ≥15.0 | *P* for trend |
| --- | --- | --- | --- | --- | --- | --- | --- |
|  | Reference | OR (95% CI) | OR (95% CI) | OR (95% CI) | OR (95% CI) | OR (95% CI) |  |
| **Baseline RDW** | | | | | | | |
| Model I | 1 | 1.18 (1.00, 1.40) | 1.36 (1.15, 1.60) | 1.65 (1.40, 1.94) | 2.08 (1.76, 2.45) | 3.39 (2.95, 3.89) |  |
| Model II | 1 | 1.15 (0.97, 1.37) | 1.30 (1.10, 1.53) | 1.56 (1.32, 1.84) | 1.95 (1.65, 2.31) | 3.20 (2.78, 3.68) |  |
| Model III | 1 | 1.17 (0.98, 1.40) | 1.30 (1.09, 1.55) | 1.60 (1.34, 1.91) | 1.92 (1.61, 2.30) | 2.92 (2.51, 3.41) | <0.0001 |
| **Mean RDW** | | | | | | | |
| Model I | 1 | 1.47 (1.19, 1.80) | 1.92 (1.58, 2.34) | 2.34 (1.93, 2.84) | 3.32 (2.74, 4.02) | 5.15 (4.34, 6.12) |  |
| Model II | 1 | 1.43 (1.16, 1.76) | 1.85 (1.52, 2.25) | 2.23 (1.83, 2.71) | 3.15 (2.59, 3.82) | 4.90 (4.12, 5.83) |  |
| Model III | 1 | 1.49 (1.20, 1.85) | 1.99 (1.62, 2.44) | 2.38 (1.94, 2.92) | 3.32 (2.71, 4.08) | 4.85 (4.04, 5.84) | <0.0001 |
| **Peak RDW** | | | | | | | |
| Model I | 1 | 1.39 (1.08, 1.80) | 1.97 (1.55, 2.50) | 2.40 (1.89, 3.04) | 3.40 (2.69, 4.29) | 5.79 (4.67, 7.18) |  |
| Model II | 1 | 1.37 (1.06, 1.76) | 1.90 (1.49, 2.42) | 2.29 (1.80, 2.90) | 3.23 (2.56, 4.09) | 5.51 (4.44, 6.83) |  |
| Model III | 1 | 1.38 (1.06, 1.79) | 1.95 (1.52, 2.50) | 2.32 (1.81, 2.97) | 3.18 (2.49, 4.06) | 5.21 (4.16, 6.53) | <0.0001 |
| **Nadir RDW** | | | | | | | |
| Model I | 1 | 1.31 (1.12, 1.54) | 1.51 (1.29, 1.76) | 1.94 (1.66, 2.27) | 2.45 (2.09, 2.87) | 3.80 (3.34, 4.33) |  |
| Model II | 1 | 1.28 (1.09, 1.50) | 1.44 (1.23, 1.69) | 1.84 (1.57, 2.15) | 2.30 (1.96, 2.70) | 3.60 (3.16, 4.11) |  |
| Model III | 1 | 1.38 (1.17, 1.63) | 1.56 (1.32, 1.84) | 2.07 (1.75, 2.44) | 2.46 (2.07, 2.92) | 3.50 (3.03, 4.05) | <0.0001 |

Model I was unadjusted. Model II was adjusted for age, sex and ethnicity. Model III was adjusted for all predefined covariates.

**eTable 6.** Univariable and multivariable adjusted odds ratios for all-cause mortality by categorical RDW with complete case analysis (n=30221)

| RDW (%) category | <13.0 | 13.0-13.4 | 13.5-13.9 | 14.0-14.4 | 14.5-14.9 | ≥15.0 | *P* for trend |
| --- | --- | --- | --- | --- | --- | --- | --- |
|  | Reference | OR (95% CI) | OR (95% CI) | OR (95% CI) | OR (95% CI) | OR (95% CI) |  |
| **Baseline RDW** | | | | | | | |
| Model I | 1 | 1.28 (1.03, 1.59) | 1.51 (1.23, 1.87) | 1.78 (1.45, 2.19) | 2.27 (1.84, 2.80) | 3.82 (3.20, 4.56) |  |
| Model II | 1 | 1.25 (1.01, 1.55) | 1.45 (1.18, 1.79) | 1.69 (1.37, 2.08) | 2.14 (1.73, 2.64) | 3.61 (3.02, 4.32) |  |
| Model III | 1 | 1.29 (1.03, 1.61) | 1.46 (1.17, 1.82) | 1.80 (1.44, 2.25) | 2.14 (1.70, 2.68) | 3.36 (2.76, 4.08) | <0.0001 |
| **Mean RDW** | | | | | | | |
| Model I | 1 | 1.56 (1.20, 2.03) | 2.04 (1.59, 2.62) | 2.62 (2.05, 3.36) | 3.58 (2.80, 4.58) | 5.73 (4.60, 7.14) |  |
| Model II | 1 | 1.52 (1.17, 1.98) | 1.96 (1.53, 2.52) | 2.50 (1.95, 3.21) | 3.42 (2.67, 4.37) | 5.46 (4.38, 6.81) |  |
| Model III | 1 | 1.60 (1.22, 2.09) | 2.13 (1.65, 2.77) | 2.81 (2.17, 3.64) | 3.57 (2.75, 4.62) | 5.46 (4.32, 6.91) | <0.0001 |
| **Peak RDW** | | | | | | | |
| Model I | 1 | 1.53 (1.10, 2.13) | 2.14 (1.57, 2.92) | 2.51 (1.85, 3.41) | 3.90 (2.89, 5.26) | 6.63 (5.03, 8.75) |  |
| Model II | 1 | 1.50 (1.08, 2.09) | 2.08 (1.52, 2.83) | 2.40 (1.77, 3.26) | 3.72 (2.75, 5.02) | 6.32 (4.79, 8.35) |  |
| Model III | 1 | 1.58 (1.13, 2.21) | 2.17 (1.58, 2.99) | 2.58 (1.88, 3.54) | 3.89 (2.85, 5.32) | 6.13 (4.58, 8.19) | <0.0001 |
| **Nadir RDW** | | | | | | | |
| Model I | 1 | 1.50 (1.22, 1.83) | 1.64 (1.35, 2.01) | 2.09 (1.72, 2.55) | 2.61 (2.14, 3.19) | 4.28 (3.62, 5.07) |  |
| Model II | 1 | 1.46 (1.19, 1.78) | 1.58 (1.29, 1.92) | 1.98 (1.62, 2.42) | 2.46 (2.01, 3.01) | 4.06 (3.43, 4.81) |  |
| Model III | 1 | 1.58 (1.28, 1.96) | 1.76 (1.42, 2.17) | 2.31 (1.87, 2.86) | 2.68 (2.16, 3.33) | 4.04 (3.36, 4.87) | <0.0001 |

Model I was unadjusted. Model II was adjusted for age, sex and ethnicity. Model III was adjusted for all predefined covariates.

**eTable 7.** Univariable and multivariable adjusted odds ratios for all-cause mortality by categorical RDW in patients with multiple measures (n=36723)

| RDW (%) category | <13.0 | 13.0-13.4 | 13.5-13.9 | | 14.0-14.4 | 14.5-14.9 | ≥15.0 | *P* for trend |
| --- | --- | --- | --- | --- | --- | --- | --- | --- |
|  | Reference | OR (95% CI) | OR (95% CI) | | OR (95% CI) | OR (95% CI) | OR (95% CI) |  |
| **Baseline RDW** | | | | | | | | |
| Model I | 1 | 1.10 (0.91, 1.32) | 1.25 (1.05, 1.50) | | 1.49 (1.24, 1.78) | 1.73 (1.45, 2.08) | 2.75 (2.37, 3.20) |  |
| Model II | 1 | 1.07 (0.89, 1.29) | 1.21 (1.01, 1.45) | | 1.42 (1.18, 1.70) | 1.65 (1.37, 1.98) | 2.62 (2.25, 3.05) |  |
| Model III | 1 | 1.07 (0.89, 1.29) | 1.21 (1.00, 1.45) | | 1.42 (1.18, 1.71) | 1.65 (1.37, 1.99) | 2.59 (2.22, 3.04) | <0.0001 |
| **Mean RDW** | | | | | | | | |
| Model I | 1 | 1.43 (1.12, 1.82) | 1.95 (1.56, 2.45) | | 2.30 (1.84, 2.88) | 3.18 (2.55, 3.97) | 4.69 (3.84, 5.73) |  |
| Model II | 1 | 1.40 (1.10, 1.79) | 1.89 (1.51, 2.37) | | 2.21 (1.77, 2.77) | 3.05 (2.44, 3.81) | 4.51 (3.69, 5.51) |  |
| Model III | 1 | 1.42 (1.12, 1.81) | 1.94 (1.54, 2.44) | | 2.28 (1.82, 2.86) | 3.15 (2.51, 3.95) | 4.62 (3.77, 5.67) | <0.0001 |
| **Peak RDW** | | | | | | | | |
| Model I | 1 | 1.27 (0.91, 1.78) | | 2.10 (1.55, 2.86) | 2.43 (1.79, 3.30) | 3.44 (2.55, 4.64) | 5.74 (4.33, 7.61) |  |
| Model II | 1 | 1.25 (0.89, 1.75) | | 2.05 (1.50, 2.79) | 2.35 (1.73, 3.18) | 3.31 (2.45, 4.47) | 5.52 (4.16, 7.32) |  |
| Model III | 1 | 1.26 (0.90, 1.76) | | 2.09 (1.53, 2.86) | 2.40 (1.77, 3.27) | 3.42 (2.52, 4.63) | 5.64 (4.24, 7.51) | <0.0001 |
| **Nadir RDW** | | | | | | | | |
| Model I | 1 | 1.29 (1.08, 1.53) | | 1.46 (1.23, 1.73) | 1.88 (1.59, 2.23) | 2.22 (1.87, 2.63) | 3.24 (2.81, 3.73) |  |
| Model II | 1 | 1.26 (1.06, 1.50) | | 1.40 (1.18, 1.67) | 1.80 (1.52, 2.14) | 2.11 (1.77, 2.50) | 3.10 (2.68, 3.57) |  |
| Model III | 1 | 1.27 (1.06, 1.51) | | 1.42 (1.20, 1.69) | 1.83 (1.54, 2.18) | 2.13 (1.78, 2.54) | 3.11 (2.68, 3.60) | <0.0001 |

Model I was unadjusted. Model II was adjusted for age, sex and ethnicity. Model III was adjusted for all predefined covariates.

**eTable 8.** Univariable and multivariable adjusted odds ratios for all-cause mortality by categorical RDW with cox proportional hazards model (n=47266)

| RDW (%) category | <13.0 | 13.0-13.4 | 13.5-13.9 | 14.0-14.4 | 14.5-14.9 | ≥15.0 | *P* for trend |
| --- | --- | --- | --- | --- | --- | --- | --- |
|  | Reference | OR (95% CI) | OR (95% CI) | OR (95% CI) | OR (95% CI) | OR (95% CI) |  |
| **Baseline RDW** | | | | | | | |
| Model I | 1 | 1.13 (0.96, 1.32) | 1.22 (1.04, 1.42) | 1.38 (1.18, 1.61) | 1.58 (1.35, 1.85) | 2.24 (1.96, 2.55) |  |
| Model II | 1 | 1.09 (0.93, 1.29) | 1.16 (0.99, 1.36) | 1.30 (1.11, 1.52) | 1.49 (1.27, 1.74) | 2.11 (1.85, 2.41) |  |
| Model III | 1 | 1.15 (0.97, 1.35) | 1.17 (1.00, 1.37) | 1.37 (1.16, 1.60) | 1.58 (1.35, 1.86) | 2.01 (1.75, 2.31) | <0.0001 |
| **Mean RDW** | | | | | | | |
| Model I | 1 | 1.37 (1.12, 1.67) | 1.62 (1.34, 1.95) | 1.81 (1.51, 2.18) | 2.29 (1.90, 2.74) | 2.92 (2.48, 3.44) |  |
| Model II | 1 | 1.33 (1.09, 1.62) | 1.54 (1.27, 1.85) | 1.71 (1.42, 2.07) | 2.15 (1.79, 2.59) | 2.75 (2.33, 3.24) |  |
| Model III | 1 | 1.39 (1.14, 1.69) | 1.63 (1.35, 1.97) | 1.80 (1.49, 2.18) | 2.29 (1.90, 2.75) | 2.71 (2.29, 3.22) | <0.0001 |
| **Peak RDW** | | | | | | | |
| Model I | 1 | 1.18 (0.93, 1.51) | 1.52 (1.21, 1.90) | 1.65 (1.32, 2.06) | 2.10 (1.69, 2.61) | 2.67 (2.18, 3.27) |  |
| Model II | 1 | 1.15 (0.91, 1.47) | 1.45 (1.16, 1.82) | 1.55 (1.24, 1.94) | 1.98 (1.59, 2.46) | 2.50 (2.04, 3.06) |  |
| Model III | 1 | 1.18 (0.92, 1.51) | 1.48 (1.18, 1.86) | 1.55 (1.23, 1.94) | 1.94 (1.55, 2.42) | 2.31 (1.88, 2.84) | <0.0001 |
| **Nadir RDW** | | | | | | | |
| Model I | 1 | 1.32 (1.13, 1.53) | 1.44 (1.24, 1.67) | 1.74 (1.50, 2.01) | 1.98 (1.71, 2.30) | 2.78 (2.45, 3.15) |  |
| Model II | 1 | 1.27 (1.09, 1.48) | 1.37 (1.18, 1.59) | 1.64 (1.41, 1.90) | 1.86 (1.60, 2.16) | 2.64 (2.32, 2.99) |  |
| Model III | 1 | 1.39 (1.19, 1.62) | 1.50 (1.29, 1.75) | 1.88 (1.62, 2.19) | 2.12 (1.82, 2.47) | 2.67 (2.34, 3.05) | <0.0001 |

Model I was unadjusted. Model II was adjusted for age, sex and ethnicity. Model III was adjusted for all predefined covariates.

**eTable 9.** Multivariable logistic regression model for all-cause mortality

| **Variable** | **OR (95% CI)** | **P value** | **Variable** | **OR (95% CI)** | **P value** |
| --- | --- | --- | --- | --- | --- |
| **Age** | 1.02 (1.01-1.02) | <0.0001 | **Age** | 1.01 (1.00-1.02) | <0.0001 |
| **Cardiac arrest or VR** | 15.64 (12.67-19.31) | <0.0001 | **Cardiac arrest or VR** | 15.45 (12.51-19.08) | <0.0001 |
| **Cardiogenic shock** | 9.00 (6.08-13.29) | <0.0001 | **Cardiogenic shock** | 8.93 (6.04-13.21) | <0.0001 |
| **ICH or SAH** | 5.14 (3.94-6.70) | <0.0001 | **ICH or SAH** | 5.37 (4.11-7.00) | <0.0001 |
| **Ischemic stroke** | 2.52 (1.97-3.23) | <0.0001 | **Ischemic stroke** | 2.60 (2.02-3.33) | <0.0001 |
| **Heart failure** | 1.96 (1.53-2.50) | <0.0001 | **Heart failure** | 1.93 (1.51-2.47) | <0.0001 |
| **PE or DVT** | 1.79 (1.22-2.61) | 0.0027 | **PE or DVT** | 1.78 (1.22-2.61) | 0.0027 |
| **Atrial arrhythmia** | 1.42 (1.05-1.92) | 0.0224 | **Atrial arrhythmia** | 1.40 (1.03-1.89) | 0.0292 |
| **Cardiac surgery** | 0.56 (0.41-0.77) | 0.0002 | **Cardiac surgery** | 0.55 (0.40-0.75) | 0.0001 |
| **Hypertension** | 0.41 (0.19-0.88) | 0.0228 | **Hypertension** | 0.39 (0.18-0.85) | 0.0170 |
| **Continuous RDW** | 1.17 (1.14-1.20) | <0.001 | **RDW 13.0-13.4** | 1.16 (0.87-1.57) | 0.3138 |
|  |  |  | **RDW 13.5-13.9** | 1.32 (0.99-1.76) | 0.0605 |
|  |  |  | **RDW 14.0-14.4** | 1.67 (1.24-2.23) | 0.0006 |
|  |  |  | **RDW 14.5-14.9** | 1.69 (1.25-2.28) | 0.0006 |
|  |  |  | **RDW ≥15.0** | 3.08 (2.40-3.95) | <0.0001 |

**eTable 10.** Multivariable logistic regression model for all-cause mortality after excluding cardiac arrest or ventricular arrhythmia and cardiogenic shock

| **Variable** | **OR (95% CI)** | **P value** | **Variable** | **OR (95% CI)** | **P value** |
| --- | --- | --- | --- | --- | --- |
| **Age** | 1.02 (1.01-1.03) | <0.0001 | **Age** | 1.02 (1.01-1.03) | <0.0001 |
| **ICH or SAH** | 5.51 (4.21-7.20) | <0.0001 | **ICH or SAH** | 5.92 (4.52-7.75) | <0.0001 |
| **Ischemic stroke** | 2.56 (1.99-3.28) | <0.0001 | **Ischemic stroke** | 2.67 (2.08-3.43) | <0.0001 |
| **Heart failure** | 1.90 (1.48-2.43) | <0.0001 | **Heart failure** | 1.84 (1.44-2.36) | <0.0001 |
| **PE or DVT** | 1.84 (1.26-2.70) | 0.0017 | **PE or DVT** | 1.83 (1.25-2.69) | 0.0018 |
| **Atrial arrhythmia** | 1.40 (1.03-1.89) | 0.0316 | **Atrial arrhythmia** | 1.36 (1.00-1.84) | 0.0467 |
| **Cardiac surgery** | 0.57 (0.42-0.77) | 0.0003 | **Cardiac surgery** | 0.55 (0.40-0.75) | 0.0001 |
| **Hypertension** | 0.46 (0.21-0.99) | 0.0465 | **Hypertension** | 0.43 (0.20-0.93) | 0.0323 |
| **Continuous RDW** | 1.20 (1.16-1.24) | <0.001 | **RDW 13.0-13.4** | 1.45 (0.97-2.13) | 0.0642 |
|  |  |  | **RDW 13.5-13.9** | 1.77 (1.21-2.58) | 0.0031 |
|  |  |  | **RDW 14.0-14.4** | 2.16 (1.48-3.15) | 0.0001 |
|  |  |  | **RDW 14.5-14.9** | 2.02 (1.35-3.01) | 0.0006 |
|  |  |  | **RDW ≥15.0** | 4.68 (3.35-6.55) | <0.0001 |
